# Standing on the shoulders of microbes: microbiome thermal priming buffers the effects of heatwaves on clams by preventing stress overreaction

**DOI:** 10.1093/ismeco/ycag059

**Published:** 2026-03-13

**Authors:** Maria Elena Martino, Marialaura Gallo, Ilaria Nai, Andrea Quagliariello, Giulia Dalla Rovere, Massimiliano Babbucci, Giovanna Monticelli, Marco Graziano, Rafaella Franch, Mbarsid Racaku, Barbara Cardazzo, Massimo Milan, Luca Peruzza, Luca Bargelloni

**Affiliations:** Department of Comparative Biomedicine and Food Science, University of Padova, 35020, Legnaro (PD), Padua, Italy; Department of Comparative Biomedicine and Food Science, University of Padova, 35020, Legnaro (PD), Padua, Italy; Department of Zoology, Genetics and Physical Anthropology, Biological Research Center (CIBUS), University of Santiago de Compostela, 15782, Santiago de Compostela, Spain; Department of Comparative Biomedicine and Food Science, University of Padova, 35020, Legnaro (PD), Padua, Italy; Department of Comparative Biomedicine and Food Science, University of Padova, 35020, Legnaro (PD), Padua, Italy; Department of Comparative Biomedicine and Food Science, University of Padova, 35020, Legnaro (PD), Padua, Italy; Department of Comparative Biomedicine and Food Science, University of Padova, 35020, Legnaro (PD), Padua, Italy; Department of Comparative Biomedicine and Food Science, University of Padova, 35020, Legnaro (PD), Padua, Italy; Department of Comparative Biomedicine and Food Science, University of Padova, 35020, Legnaro (PD), Padua, Italy; Department of Comparative Biomedicine and Food Science, University of Padova, 35020, Legnaro (PD), Padua, Italy; Department of Comparative Biomedicine and Food Science, University of Padova, 35020, Legnaro (PD), Padua, Italy; Department of Comparative Biomedicine and Food Science, University of Padova, 35020, Legnaro (PD), Padua, Italy; Department of Comparative Biomedicine and Food Science, University of Padova, 35020, Legnaro (PD), Padua, Italy; Department of Comparative Biomedicine and Food Science, University of Padova, 35020, Legnaro (PD), Padua, Italy; Department of Comparative Biomedicine and Food Science, University of Padova, 35020, Legnaro (PD), Padua, Italy

**Keywords:** thermal stress, heat waves, microbiome, host–microbe interactions, thermal priming, bivalves

## Abstract

In the context of rapidly accelerating global warming, the rising frequency of heatwaves is driving large-scale ecological shifts, profoundly affecting organismal physiology and ecosystem functioning. Thermal tolerance is a key determinant of species resilience. Evidence from diverse model systems indicates that this tolerance can be enhanced through thermal priming, a pre-adaptive process in which organisms are exposed to sublethal heat stress. Beyond intrinsic host factors, the adaptive potential of host-associated microbiomes is increasingly recognised as a critical role in shaping organismal thermal resilience. However, the extent to which microbiomes alone can enhance host thermal tolerance remains largely unknown. Here, we used the Manila clam (*Ruditapes philippinarum*), one of the most widely farmed bivalves, as a model system to test whether thermal pre-adaptation of the microbiome is sufficient to improve host thermal tolerance. Clams were thermally primed, their microbiota isolated, and subsequently transplanted into non-acclimated individuals, which were then exposed to simulated heatwave conditions. By integrating microbial community profiling, host physiological measurements, and transcriptomic analyses, we demonstrate that transplantation of a microbiome from animals previously exposed to heat stress is sufficient to enhance host resilience during subsequent heat stress. This effect arises from adaptive shifts in microbiome composition that promote energy conservation and survival through elevated antioxidant activity and a broad downregulation of host transcriptional pathways, placing the host in an energy-efficient, stress-mitigating state. Our findings provide novel insights into holobiont-level adaptive mechanisms to stress adaptation and hold practical potential for developing microbiome-based interventions to enhance thermal tolerance in aquaculture systems.

## Introduction

We are witnessing an era of unprecedented global environmental change. Over the past 15 years, human activities have pushed six of the nine planetary boundaries beyond safe operating limits [1]. Against this backdrop, global warming is accelerating. In marine environments, the increasing frequency of heatwaves (i.e. periods when daily sea surface temperatures exceed the 90th percentile of climatological records for more than five consecutive days [2]), have already been directly linked to widespread mass mortality events affecting a broad spectrum of marine organisms, including fish, molluscs, corals, and even seabirds [3–10]. The consequences have extended across diverse habitats, from open-ocean to coastal ecosystems, including aquaculture systems, which hold significant ecological and socio-economic value [11].

Ectotherms are especially vulnerable to warming, as their body temperatures fluctuate with external conditions [12, 13]. They rely heavily on access to suitable microclimates and behavioral thermoregulation, such as seeking shade or burrowing, to buffer against heat stress. However, prolonged exposure to elevated temperatures increases metabolic demands and energy expenditure, ultimately reducing fitness and, in severe cases, leading to mortality [14, 15]. In this context, thermal tolerance—an organism’s capacity to withstand variations in temperature—is a key determinant of species ecology and evolutionary potential [16]. It is shaped by a multitude of factors, including genetic background and regulation, developmental stage, and external environmental conditions [17]. Moreover, it has been documented that thermal priming (i.e. exposure to sublethal temperature stress) can enhance organism resilience to future heat events by inducing a form of stress memory [18–20].

Beyond the host’s intrinsic responses to heat stress, microbiomes are increasingly recognized as critical contributors to buffering animals against temperature fluctuations [21, 22]. Microbial communities quickly adapt to environmental change, due to their large population sizes and rapid metabolic and growth rates. Their responses occur at multiple levels: at the cellular scale, through physiological plasticity and regulation of gene expression, and at the community scale, via ecological processes such as species turnover [23–25]. This complex interplay of mechanisms is central to what is termed *microbiome flexibility*, the ability of a host’s microbial community to dynamically restructure in response to changing environmental conditions [26], and as such, is now regarded as a fundamental phenomenon that enhances host resilience to thermal stress [16, 27–29]. The structure and function, alongside compositional shifts, of host-associated microbial communities have been identified as key drivers of thermal tolerance and metabolic regulation in animals exposed to elevated temperatures [30–32]. Under such conditions, functionally resilient or responsive microbiomes are critical to host health and survival [33–35].

Within this context, microbiome-mediated acclimation to elevated temperatures may play an active role in shaping animal thermal tolerance. This is particularly relevant for ectothermic organisms. For example, corals such as *Acropora hyacinthus* harbor heat-tolerant microbial strains that improve survival under thermal stress [36]. In amphibians, depletion of gut microbiome diversity led to reduced thermal tolerance, resulting in decreased survival during acute heat stress [37]. Microbiota transplants from heat-tolerant individuals to more sensitive hosts have demonstrated the potential to improve thermal resilience across diverse taxa, including cnidarians, flies and mice [34,38,39].

Despite these findings, the extent to which host resilience can be enhanced solely through microbiome-mediated acclimation, and the underlying mechanisms, remain largely unknown. Understanding these dynamics is particularly important in the current context, as microbiome-based interventions are becoming increasingly popular across diverse organisms. This study aims to test whether microbiome-mediated acclimation is sufficient to increase host thermal tolerance and to identify the specific holobiont-level mechanisms involved. To address this, we employed the Manila clam (*Ruditapes philippinarum*) as a model system. *R. philippinarum* is one of the most widely farmed molluscs globally and plays important ecosystem functions in controlling and regulating water turbidity. Further, *R. philippinarum* has been long studied in the context of environmental pollution and climate change using integrative biological approaches [40–42]. Recently, we demonstrated that thermal priming enhances clam resilience to lethal heatwaves compared to naïve individuals [20]. In this study, we thermally primed clams, isolated their microbiota and transplanted it into non-acclimated clams, which were then exposed to a simulated heatwave. By integrating microbial community composition, host physiological traits, and gene expression data, we demonstrate that transplantation of a microbiome from animals previously exposed to heat stress is sufficient to enhance host resilience during subsequent heat stress and identify holobiont-level mechanisms underlying this effect. Altogether, our findings demonstrate that the transmission of acclimated microbial communities may serve as a mechanism for host rapid adaptation to elevated temperatures.

## Materials and methods

### Clam maintenance

Samples of Manila clam (*Ruditapes philippinarum*) (n = 200, average length = 13.01 ± 1.56 mm, average total weight = 3.024 ± 0.52 g) were purchased from the SATMAR hatchery (France). Upon arrival, clams were placed in a 20 L aquarium containing artificial seawater (ASW, Aquaforest Sea Salt) adjusted to a salinity of 33 PSU and maintained at room temperature for 1 h. Subsequently, clams were transferred to a 100 L aquarium and acclimated at 25° C for 5 days. Aeration was provided continuously with air stones to ensure adequate oxygenation. During acclimation, clams were fed once every two days with 10 ml/tank of New Coral Fito Concentrate (A.G.P., Italy), a commercial microalgal blend consisting of *Isochrysis* (T-Iso) (33.3%), *Nannochloropsis* (31%), *Tetraselmis* (18%), and *Phaeodactylum* (18%), at a final concentration of 10^9 cell/ml. Approximately 50% of the aquarium water was replaced twice during the acclimation period with freshly prepared ASW pre-equilibrated to the same temperature.

### Experimental design

#### Microbiota thermal priming

**Figure 1:**
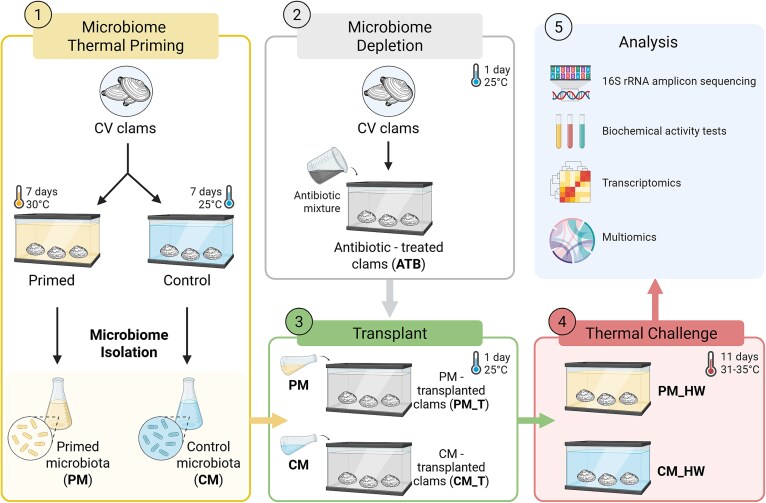
Experimental setup. (A) Conventional (CV) clams were acclimated and then divided into two thermal treatments: priming at 30° C for 7 days or control at 25° C for 7 days. At the end of this period, the total-body microbiome was isolated from clams in each group. (B) In parallel, antibiotic-treated clams (ATB) were produced to generate microbiome-depleted recipients for transplantation. (C) The isolated microbiomes from primed and control clams (PM and CM, respectively) were used to inoculate the respective groups of ATB clams during transplantation. (D) Following microbiome transplantation, clams were exposed to a thermal challenge with water temperature fluctuating between 31° C and 35° C. (E) At the end of the experiment, clams were dissected, and tissues were collected for physiological, transcriptomic, enzymatic, and microbiota analyses. Sample abbreviations are defined in Table 1.

After 5 days of acclimation, 126 clams were randomly distributed into six tanks: three *Priming* tanks, where clams were maintained for 7 days at 30° C, and three *Control* tanks, where clams were kept for 7 days at 25° C. At the end of the priming period, the whole bodies of 12 clams from each replicate tank were homogenized in Phosphate-Buffered Saline (PBS) (P4417, Sigma-Aldrich) using a QIAGEN TissueLyser II, resulting in six homogenates (three from the primed microbiota (PM) group and three from the control microbiota (CM) group) (Fig. 1). Each homogenate was sequentially filtered: first through a coarse mesh filter to remove large debris, followed by filtration through a 100 μm Corning Cell Strainer. The final filtered homogenates were plated out on Marine Agar medium (Condalab, Spain) to assess bacterial abundance after treatment, which was found to be ~10^6^ CFU ml^−1^ each. The homogenates were then supplemented with 30% (v/v) of 80% glycerol and stored at −80° C until further use.

#### Microbiome transplantation

Before transplantation, antibiotic-treated clams (ATB) were prepared following the protocol described by Gallo et al. [43]. After 5 days of acclimation, 108 clams were distributed into six tanks containing 3 L of ASW at 25° C. One hour later, the first dose of antibiotics was administered. 24 h after antibiotic treatment, ATB clams were randomly assigned to three replicate groups per treatment (PM_T and CM_T), with 15 clams placed in each small tank containing 0.5 L of ASW. The previously isolated microbiota (PM and CM) was then added to the respective tanks along with feed to promote microbiome uptake by the clams. After 1 h, the clams and the 0.5 L of ASW were transferred to larger tanks containing an additional 2.5 L of ASW (final volume 3 L).

#### Thermal challenge

24 h post-transplantation, the ASW was replaced with fresh ASW and the tanks were then subjected to a thermal challenge for 11 days, during which water temperature oscillated between 31° C and 35° C, following the protocol described by Peruzza et al. [40]. Mortality was monitored daily throughout the challenge.

Table 1 summarizes the sample types and analyses performed for each experimental group.

**Table 1:** Description of sample abbreviations used in the study and the corresponding analyses performed.

**Abbreviation**	**Description**	**Analysis performed**
ATB	Antibiotic-treated clams (24 h at 25° C)	16S rRNA sequencing (whole body)
PM	Microbiome isolated from the whole body of clams following thermal priming (7 days at 30° C)	16S rRNA sequencing (whole body)
CM	Microbiome isolated from the whole body of control clams (not subjected to thermal priming; maintained for 7 days at 25° C)	16S rRNA sequencing (whole body)
PM_T	Microbiome isolated from the whole body of clams 24 h after transplantation with primed microbiota (PM)	16S rRNA sequencing (whole body)
CM_T	Microbiome isolated from the whole body of clams 24 h after transplantation with control microbiota (CM)	16S rRNA sequencing (whole body)
PM_HW	Clams previously transplanted with primed microbiota (PM) and subsequently exposed to heatwave conditions for 11 days at 31–35° C. For this group, the microbiome was isolated from the digestive gland	16S rRNA sequencing (digestive gland)
		Survival
		Host RNA sequencing (digestive gland)
		Enzymatic assays (mantle)
		Multiomics
**CM_HW**	Clams previously transplanted with control microbiota (CM) and subsequently exposed to heatwave conditions for 11 days at 31–35° C. For this group, the microbiome was isolated from the digestive gland	16S rRNA sequencing (digestive gland)
		Survival
		Host RNA sequencing (digestive gland)
		Enzymatic assays (mantle)
		Multiomics

### RNA extraction

For RNA extraction, either the previously stored pellet or a portion of the digestive gland (<30 mg) was used. For the ATB, PM, CM, PM_T, and CM_T experimental groups, pellets were obtained by homogenizing the whole body of three clams from each tank in 9× Phosphate-Buffered Saline (PBS) (P4417, Sigma-Aldrich) using a Stomacher 3500 (VWR, Italy) for 2 min at 400 rpm (maximum speed). Following homogenization, 2 ml of the filtrate was centrifuged at 12000 rpm for 5 min; the supernatant was discarded, and the resulting pellet was stored on dry ice at −80° C. For the PM_HW and CM_HW experimental groups, the digestive gland was dissected for microbiome analysis, as it was the chosen tissue for host transcriptomics, allowing for a more straightforward comparison between omics datasets. In both cases, either from pellets or dissected tissue, 0.5 μm glass beads were added, and RNA was extracted using the RNeasy Mini Kit (Qiagen, Germany) according to the manufacturer’s instructions. RNA purity, concentration, and integrity were assessed using NanoDrop ND-1000 spectrophotometer (Thermo Scientific, USA) and Bioanalyzer 2100 system (Agilent Technologies, USA). The extracted RNA was used for both host gene expression profiling and microbiota characterisation (16S rRNA sequencing).

### 16S rRNA sequencing and analysis

For 16S rRNA gene sequencing, 1 μg of total RNA was reverse-transcribed into cDNA using the SuperScript™ IV First-Strand Synthesis System (Invitrogen™, USA). RNA was targeted, rather than DNA, to selectively analyze active bacteria and avoid the inclusion of genetic material from dead cells. Library preparation and sequencing of the V3–V4 hypervariable regions (338F: 5’-ACTCCTACGGGAGGCAGCA-3′; 806R: 5′-GGACTACHVGGGTWTCTAAT-3′) of the bacterial 16S rRNA gene were carried out by BMK Gene (Germany). Sequencing was performed on an Illumina NovaSeq 6000 platform with a paired-end 250 bp. Raw sequencing data were processed to remove adapter sequences, filtered by read length, and low-quality reads were eliminated using Fastp [44], Trimmomatic v0.33 [38], and Cutadapt 2.7.8 [39]. The cleaned reads were then analyzed in R using the dada2 package [45] to infer Amplicon Sequence Variants (ASVs) and assign taxonomy based on the SILVA reference database (silva_nr99_v138.1_wSpecies_train_set.fa).

Biodiversity metrics were calculated using the phyloseq [46] and *vegan* packages in R. For alpha diversity metrics, statistical comparisons among groups were performed using nonparametric Kruskal–Wallis tests followed by Dunn’s post-hoc tests to evaluate selected pairwise contrasts of biological interest. Multiple testing correction was applied only across these predefined contrasts using the Benjamini–Hochberg false discovery rate (FDR) procedure. Bray–Curtis dissimilarity was used to assess beta diversity, and the effects of treatment variables (treatment, time, temperature—see Table S1 for details and categories) were tested using the *Adonis2* function (PERMANOVA). DESeq2 was used to identify differences in bacterial taxa between experimental groups, and the significantly different genera were visualized in a heatmap based on their relative abundance [47].

### RNA-seq analysis

RNA-seq libraries preparation was carried out by BMK Gene (Germany) and sequenced on an Illumina NovaSeqX platform with a paired-end 150 bp setup. Sequencing reads were processed using the nf-core/rnaseq pipeline v3.18 [48], a Nextflow-based, modular pipeline for reproducible RNA sequencing analysis. The pipeline was executed on a Linux-based high-performance computing cluster using Nextflow v25.04.4 and Singularity for containerization to ensure reproducibility and consistency of the computational environment.

Quality control metrics, including sequence quality, duplication rates, and alignment statistics, were summarized using MultiQC v1.25. Contaminants, adapter content, and rRNA content were also evaluated during preprocessing. Reads were aligned to the *R. philippinarum* genome (Peruzza et al., in preparation) using STAR v2.7.10a, and transcript quantification was performed using RSEM v1.3.1. Gene-level count file was generated and imported to R for downstream differential expression analysis.

To reduce noise, genes with fewer than five reads in more than 30% of samples were filtered out, retaining 15 525 genes. Count data were normalised using the “RUVs” function in the RUVSeq v 1.42.0 package with k = 2. Differential expression (DE) analysis was conducted using edgeR v 4.6.2. A likelihood ratio test was performed with “glmFit” and “glmLRT,” and genes with a false discovery rate (FDR) < 0.05 were considered significantly differentially expressed.

Functional annotation and enrichment analysis of DEGs were performed using clusterProfiler v 4.16.0. Clam gene transcripts were annotated by sequence similarity to the Ensembl human proteome using BLASTp [49] as described in Peruzza et al. [40]. Pathway analysis was performed by means of Gene Set Enrichment Analysis (GSEA) [50]. Pathways with FDR < 0.05 were considered significant. Human functional databases from the Gene Ontology and KEGG consortium were downloaded from gProfiler (https://biit.cs.ut.ee/gprofiler/gost) and used as input for GSEA.

Visualisations, including heatmaps of DEGs, were generated with ComplexHeatmap v2.14, while PCA plots, bar plots, dot plots and emapplot of the GSEA results were created using libraries enrichplot v 1.28.2 and ggplot2 v3.4.

### Enzymatic assays and survival analysis

Enzymatic activities of Superoxide Dismutase (SOD), Catalase (CAT), Glutathione Peroxidase (GPx), Lipid Peroxidation (LP), and total protein content (BCA) were assessed on ~10 mg of mantle tissue dissected from clams exposed to heat waves (PM_HW, CM_HW). Mantle tissues were flash-frozen in liquid nitrogen and stored at −80° C until analysis. For SOD, CAT, and GPx assays, each tissue sample was homogenised using a QIAGEN TissueLyser II in 300 μl of homogenisation buffer composed of 150 mM NaCl, 1 mM EDTA, 1 mM EGTA, 5% Nonidet, 0.5% PIC200X, 1% Phosphatase Inhibitor Cocktail, 0.1% Triton X-100, and 10 mM Tris–HCl. Samples were then centrifuged at 10 000 × g for 20 min at 4° C, and the resulting supernatants were aliquoted and stored at −80° C until further analysis.

Total protein content was measured using the Pierce BCA Protein Assay Kit (Thermo Fisher Scientific, Cat. No. A55865), following the manufacturer’s instructions. Enzymatic activities were subsequently normalised to the total protein content of the corresponding sample.

SOD activity was quantified using the Superoxide Dismutase Activity Assay Kit (CS0009, Sigma-Aldrich) on a 96-well plate, according to the manufacturer’s protocol. Results were expressed as U/mg protein, where one unit corresponds to the dismutation of one micromole of superoxide anion into molecular oxygen and hydrogen peroxide per minute.

CAT activity was determined using the Catalase Assay Kit (CAT100, Sigma-Aldrich), performed in 1 ml cuvettes following the manufacturer’s instructions. Activity was expressed as U/mg protein, where one unit represents the amount of enzyme that decomposes 1 μmol of hydrogen peroxide per minute.

GPx activity was measured by mixing 25 μl of each sample (composed of 12.5 μl sample in homogenisation buffer and 12.5 μl dilution solution: PBS 6.25×, EDTA 6.25 mM) with 80 μl of assay solution containing 20 mM NADPH, 130 mM reduced glutathione (GSH), and 889.2 U/ml glutathione reductase (GR). The reaction was initiated by adding 20 μl of cumene hydroperoxide assay solution (82.2% dilution solution, 52.6 mM cumene hydroperoxide). Absorbance at 340 nm was recorded twice, with a 5-min interval. GPx activity was calculated as the rate of NADPH consumption, expressed as nanomoles of NADPH consumed per minute per mg of protein.

For LP measurement, each sample was homogenised in 300 μl of MDA Lysis Buffer supplemented with 3 μl of BHT, using the QIAGEN TissueLyser II. LP levels were then quantified with the Lipid Peroxidation (MDA) Assay Kit (MAK568, Sigma-Aldrich) on a 96-well plate, following the manufacturer’s protocol. Results were expressed as nanomoles of malondialdehyde (MDA) per mg of tissue. For each quantified parameter, normality was assessed using the Shapiro–Wilk test. When data were normally distributed, a Student’s t-test was applied; for non-normally distributed data, a non-parametric Mann–Whitney test was used. Differences in survival between treatments (PM_HW *vs* CM_HW) were analyzed using the Log-Rank (Mantel–Cox) test.

### Integrative multi-omics analysis

To explore cross-omics associations between 16S rRNA sequencing data (microbiome) and host RNA-seq data (transcriptome), we applied the Data Integration Analysis for Biomarker discovery using Latent cOmponents (DIABLO) framework [51] on PM_HW and CM_HW clam data. Prior to integration, dimension reduction was performed using an information gain algorithm to retain only the most informative features within each dataset. Data integration and modeling were conducted using the DIABLO module of the MixOmics R package. A sparse Partial Least Squares Discriminant Analysis (sPLS-DA) model was used to assess correlations between omics layers within each group. The tune.block.splsda function was used to optimize the parameters of the sPLS-DA model. To identify the most discriminative features across the transcriptome and microbiome datasets, a parameter tuning grid was applied, testing a range of variable selection values between 20 and 150 for each data block. Cross-validation was performed to select the optimal number of components and variables to retain per block, based on the minimization of the balanced error rate (BER). This approach ensured robust feature selection while accounting for the heterogeneity of the input datasets. Following model validation, circle correlation plots were generated to visualize the associations between 16S rRNA sequencing and transcriptomic features, as implemented in the DIABLO package.

## Results

The experimental design we developed is illustrated in Fig. 1. In detail, we first acclimated adult conventional (CV) clams, which were then either subjected to a sublethal thermal stress (thermal priming; 7 days at 30° C) or maintained at a control temperature (7 days at 25° C). After this period, we isolated the microbiome from both treatments (PM and CM) and used it to inoculate naïve clams whose native microbiota had been depleted using an antibiotic mixture. Following microbiome transplantation, the clams were exposed to a simulated heatwave stress (31–15 ° C for 11 days).

### Thermal priming induces distinct microbiome shifts in challenged clams

**Figure 2:**
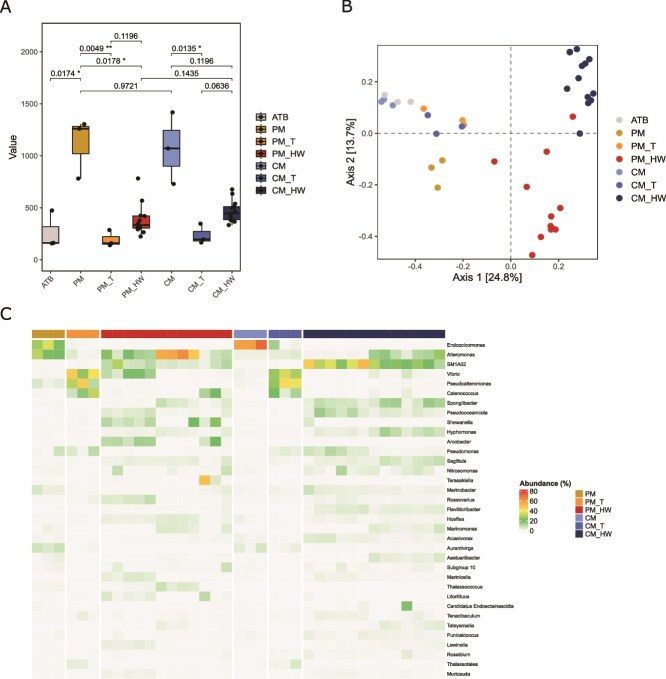
(A) Comparison of α-diversity between analysed samples. Box plots show the observed diversity indices for each experimental group. Statistical comparisons were performed using the Kruskal–Wallis test followed by Dunn’s post-hoc pairwise comparisons limited to the selected biologically relevant contrasts. Adjusted *P*-values were corrected for multiple testing using the Benjamini–Hochberg (FDR) method. Box plots display the median (center line), the first and third quartiles (box limits), and whiskers extending to 1.5 × the interquartile range. Adjusted *P*-values are provided for the statistical comparisons between groups (B) principal coordinates analysis (PCoA) at the ASV level based on Bray–Curtis dissimilarity, illustrating sample clustering according to treatment. (C) Heatmap showing variation across samples for bacterial genera identified as significant by DESeq2 analysis. Sample abbreviations are described in Table 1.

We first analyzed the microbiome composition of all experimental groups through 16S rRNA amplicon sequencing to evaluate treatment-related shifts in the microbial communities. Samples were sequenced on the Illumina NovaSeq X platform. After quality control, assembly, and data filtration, a total of 8 445 209 clean reads were obtained. The number of clean reads per sample ranged from 94 258 to 544 669, with an average of 211 130 clean reads per sample (Table S1). PERMANOVA analysis showed that treatment (defined as the combined effect of microbiome transplantation and thermal protocol) significantly explained microbiome composition (R^2^ = 0.17; *P* = .001). Alpha diversity analysis revealed no significant differences between antibiotic-treated clams and microbiome-transplanted clams. In contrast, both groups exhibited significantly reduced alpha diversity compared with thermally primed and control clams when assessed using the Observed diversity index (Fig. 2A). The Shannon index showed a significant difference only between PM and PM_T (Fig. S1). Following microbiome transplantation, exposure to thermal challenge led to a slight increase in the number of observed taxa; however, this increase was not significant according to the Observed diversity index (Fig. 2A) and was significant only for the comparison between CM_T and CM_HW when community evenness was taken into account (Fig. S1). To evaluate the impact of treatment on the composition of the microbial community, we performed a principal coordinates analysis (PCoA). Thermal priming alone induced a significant shift in the host microbiome compared with control clams (Figs. 2B, S2), while microbiome transplantation resulted in partial convergence of microbial communities (Fig. 2B, S3). Nevertheless, both PCoA axes effectively distinguished thermal-challenged clams from all other treatment groups (Axis 1). This may be due, at least in part, to differences in the tissues analyzed (i.e. digestive gland for PM_HW and CM_HW versus whole body for the remaining groups). At the same time, Axis 2 significantly separates the PM_HW and CM_HW microbiomes. More specifically, while only one genus (*Marinomonas*) was significantly differentially abundant between PM_T and CM_T (Fig. S4B), thermally challenged clams exhibited 53 genera with significant differential abundance between treatments, including *Vibrio, Alteromonas, Shewanella, Aequerovita, Tepidibacter*, and *Roseovarius* (Fig. 2C; Fig. S4C).

### Transplantation of thermally primed microbiota increases heat stress tolerance

**Figure 3:**
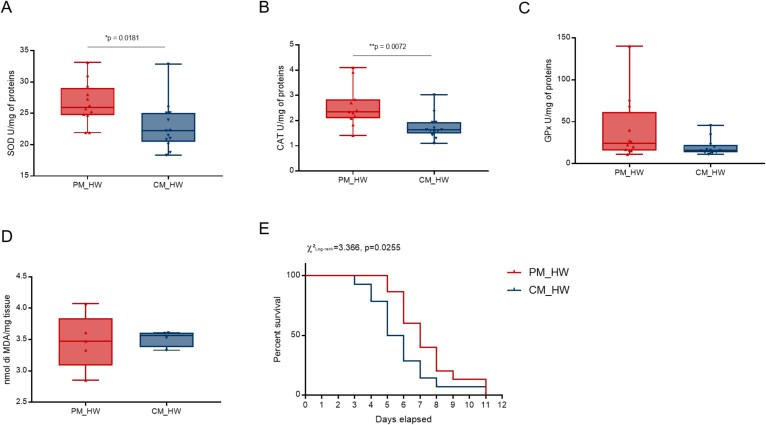
Impact of thermal challenge on physiological traits and biochemical activities of clams after microbiome transplantation. (A–D) Panels show the effect of heat stress on superoxide dismutase activity (SOD, A), catalase activity (CAT, B), glutathione peroxidase activity (GPx, C), and lipid peroxidation levels (malondialdehyde—MDA, D). Statistical significance (determined using Student’s t-test) is indicated within each figure. (E) Kaplan–Meier survival curve is presented for survival analysis. Statistical details (Log-Rank Mantel-Cox test) are reported in the figure.

We next analyzed the physiological response of clams transplanted with thermally primed microbiota and subjected to thermal stress by assessing host oxidative stress response and monitoring survival in both experimental groups (PM_HW and CM_HW). At the biochemical level, PM_HW clams exhibited significantly higher activities of the antioxidant enzymes superoxide dismutase (SOD) and catalase (CAT) compared to controls (Fig. 3A and B). In contrast, glutathione peroxidase (GPx) activity and lipid peroxidation levels did not differ significantly, although GPx showed a trend toward higher activity in PM_HW clams (Fig. 3C and D). Notably, clams transplanted with the thermally primed microbiome and exposed to heatwave conditions (PM_HW) exhibited enhanced resistance to heatwave stress, as reflected by a significantly prolonged survival time compared to CM_HW clams (*P* = .0255, Fig. 3E).

**Figure 4:**
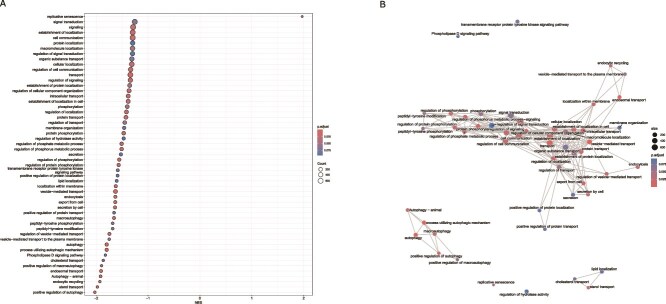
Transcriptomic response of clams exposed to heatwave conditions after receiving the thermally primed (PM_HW) versus control microbiome (CM_HW). (A) Gene Set Enrichment Analysis (GSEA) of host transcriptomic response to microbiome transplantation and thermal challenge. Dot plot showing enriched Gene Ontology (GO) biological processes between clams transplanted with thermally primed microbiomes (PM_HW) and control microbiomes (CM_HW), both exposed to heatwave conditions. The x-axis represents the normalized enrichment score (NES), with negative values indicating gene sets downregulated in PM_HW clams. Dot color reflects the adjusted *P*-value, and dot size corresponds to the number of genes contributing to each gene set. (B) Functional enrichment network of GO biological processes obtained through GSEA. Each node represents a gene set, with node color indicating statistical significance (adjusted *P*-value) and node size reflecting the number of genes within each set. Edges represent shared genes between gene sets, with edge thickness proportional to the number of overlapping genes.

RNA-seq analysis on digestive gland tissues showed only seven differentially expressed genes (DEGs) between PM_HW and CM_HW groups (Table S2, Fig. S5). Gene Set Enrichment Analysis (GSEA) identified 52 enriched pathways in PM_HW clams, of which 51 were down-regulated and 1 was up-regulated. These included pathways associated with autophagy, cholesterol and sterol metabolism, vesicle-mediated transport, and phosphorylation (Fig. 4A). Replicative senescence was the only gene set found to be upregulated in clams receiving the thermally primed microbiome. To visualize the broader functional architecture of these transcriptomic changes, we constructed a functional enrichment network. This revealed three main clusters of interconnected pathways, all downregulated in PM_HW clams (Fig. 4B): a central, highly interconnected cluster centered around phosphorylation, signal transduction, cell communication, and vesicle-mediated protein transport; a distinct module encompassing six pathways involved in autophagy processes, and a third cluster enriched in lipid metabolism pathways, such as cholesterol and sterol transport. Further analysis revealed that the genes contributing most to the enrichment and downregulation of the sterol pathway in PM_HW clams are predominantly involved in cholesterol and sterol transport, and especially lipid localization (Fig. S6A). Key drivers of this downregulated signature include *TMEM30A, BLTP3B*, and *CDS2*, which are associated with lipid localization, as well as multiple members of the ATP-binding cassette (ABCA) transporter family (e.g. *ABCA1, ABCA3, ABCA5*), the OSBP/OSBPL family, and additional genes such as *EEPD1, STARD3*, and *LAMTOR1*, all of which contribute to the three pathways (Fig. S6A). A comprehensive downregulation was observed in genes associated with crucial autophagy functional modules in PM_HW clams. This included genes involved in membrane trafficking (*WDR45, ATG101, RAB39B*), organelle-specific degradation (*BNIP3, FUNDC1, PEX5*) and lysosomal and mitochondrial quality control (*TPCN1, CTSK, SPTLC1*) (Fig. S6B). The downregulation affected both upstream regulators and downstream effectors, possibly indicating a global suppression of the autophagic cascade in clams that received the thermally primed microbiome.

### Microbiome acclimation is tightly associated with host transcriptomic regulation

**Figure 5:**
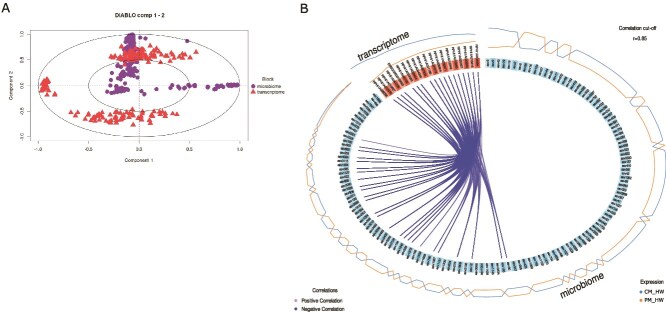
(A) Correlation circle plot on the two datasets. Variable types are indicated with different symbols and colours, and are overlaid on the same plot. (B) Circos plot generated from the DIABLO integrative analysis illustrating positive and negative correlations between host transcriptomic features and 16S rRNA microbiome features on the first component, using an absolute correlation cutoff of 0.85. Each connecting link represents a significant correlation between a gene and an ASV (amplicon sequence variant), with blue line color indicating the direction of the association (negative). Feature expression profiles are shown as continuous tracks: Orange lines represent relative abundance in PM_HW clams, while blue lines represent abundance in CM_HW clams.

We next assessed the extent to which microbiome acclimation correlates with the host transcriptomic response using a supervised multi-omics integrative analysis designed to maximize correlations between different omics layers and identify key traits that discriminate between sample groups. This approach was applied to investigate the relationship between 16S rRNA microbiome profiles and host RNA-seq data from PM_HW and CM_HW clams. The extracted components from each dataset exhibited a strong correlation (r = 0.92), highlighting a good agreement between the microbiome and transcriptome datasets (Fig. S7). Most microbiome variables were positively correlated with both components, whereas transcriptomic variables were predominantly negatively correlated with the first component and separated mainly along the second (Fig. 5A). The Circos plot of correlations for the first component revealed exclusively negative associations between microbial and host transcriptomic features, with 26 ASVs significantly negatively correlated with 18 host genes (Figs 5B, S8, Tables S3–S5). No significant correlations were observed on the second component. Among the characterized genes most influential in defining the shared component (i.e. those with larger absolute loadings) were genes involved in stress-response regulation (e.g. RPHI1A015965, RPHI1A003358, RPHI1A025097) and the degradation of complex carbohydrates for energy release (e.g. RPHI1A004984; Fig. S8).

## Discussion

In this study, we demonstrate that microbiome-mediated acclimation is sufficient to enhance host thermal tolerance, even in the absence of host preconditioning. This increased tolerance appears to be driven by adaptive shifts in microbiome composition, which leave legacy effects, ultimately improving energy conservation and host survival. This occurs through enhanced antioxidant activity and the broad downregulation of host transcriptional pathways, which place the host in an energy-conserving state and mitigate the harmful effects of excessive stress responses.

Thermal stress is mainly known to impair microbiome diversity [16,52–56]. However, responses to high temperature can vary depending on whether holobionts are adapted to fluctuating or stable thermal environments. Some studies have even shown that moderate increases in temperature may enhance microbial diversity, as higher temperatures can activate dormant bacteria [57–59], allowing them to replicate and become detectable. In our study, the thermal challenge following microbiome transplantation resulted in a slight increase in alpha diversity in both experimental groups (Fig. 2A), although this difference was not statistically significant. This pattern is likely shaped both by the earlier antibiotic treatment—which substantially reduced microbial diversity relative to the non-treated groups, and by the fact that, in challenged clams, the microbiome was sampled from the digestive gland rather than the whole body.

In addition to differences in overall diversity, we observed significant compositional changes across all treatment groups. Thermal priming of the microbiomes imposed a strong selective pressure that drove ecological shifts and generated legacy effects that remained detectable after the thermal challenge, with PM_HW clams exhibiting significantly higher abundances of several key genera compared with their CM_HW counterparts. Some of these genera, such as *Alteromonas*, were already differentially abundant in the PM group prior to heat exposure, whereas others (e.g. *Shewanella, Thalassococcus*, and *Vibrio*) were detected only after the thermal challenge (Fig. S4). These genera are primarily members of the Alphaproteobacteria and Gammaproteobacteria, two bacterial classes that have frequently been associated with improved thermal tolerance in marine invertebrates. In particular, Alphaproteobacteria are known for their antioxidant capabilities and play a protective role in coral holobionts under thermal stress [60,61]. Alongside, Gammaproteobacteria, such as *Alteromonas* strains, *V. coralliilyticus* and *V. shiloi*, have also been shown to inhibit pathogens and increase heat tolerance in corals [62,63] and supply essential nutrients [64–66].

It is important to note that 16S rRNA gene sequencing data are based on relative rather than absolute abundances, which limits their ability to reflect true microbial cell counts within each sample and complicates direct comparisons among samples. In addition, microorganisms harboring multiple copies of the 16S rRNA gene may appear more abundant than they are in reality. Furthermore, although the only difference between our challenged clam groups was the transplanted microbiome, our results make it difficult to determine whether the observed differences in the abundance of specific microbial taxa directly benefit the host and what the temporal effects are. These shifts likely represent a combination of host-directed and microbe-centred responses to thermal stress. Our findings show that, although microbiome-transplanted clams initially appear to share similar community compositions, thermally preconditioning the microbiome seems to impart an intrinsic thermal legacy that persists beyond taxonomic similarity, which ultimately profoundly influences the host’s physiological response and adaptation. Bacteria that have previously been exposed to heat may also retain traits or functional capacities that continue to benefit the host when subjected to subsequent stress. Indeed, it is worth noting that compositional similarity primarily refers to taxonomic identity, yet functional variability can persist even among similar or identical taxa. This functional plasticity is likely to be a key component of the observed improvements in host thermal resilience and can be further understood by shifting focus to the host’s response to these microbiomes.

Symbionts can promote host thermal tolerance through a variety of mechanisms. While mechanisms of specific bacterial species start to be unravelled [67], the role of complex microbiomes remains less understood. In our study, we found that transplanting microbiomes from thermally acclimated donors was sufficient to enhance host thermal resilience and significantly prolonged survival (Fig. 3E). Mechanistically, this was accompanied by elevated antioxidant enzyme activity and global downregulation of host transcriptional pathways involved in stress and energy-demanding cellular processes (Figs 3–5). Such energy conservation strategies are crucial for ectothermic organisms, which usually depend on intrinsic metabolic adaptations, the clearance of reactive oxygen species (ROS) and the regulation of genes linked to heat shock and oxidative stress in order to cope with thermal challenges. For example, marine invertebrates enter a hypometabolic state, characterised by reduced metabolic activity and heart rate, to minimise energy expenditure when exposed to extreme heat [68,69]. In our experiment, the increased activity of antioxidant enzymes observed in clams harbouring acclimated microbiomes suggest a microbiome-mediated protective effect that could mitigate oxidative damage and help maintain energetic homeostasis during heat stress.

A key question emerging from these results is the causal origin of the enhanced oxidative response: is it a product of coordinated host-microbiome activity in response to environmental stress, or is it primarily a host-driven reaction to both thermal exposure and specific microbial cues? Notably, in amphibians, the presence of a microbiome has been linked to increased production of reactive oxygen species during heat stress, and the elevated abundance of these microbial functions may contribute to improved heat tolerance [37]. Clarifying these relative contributions will be crucial for understanding the mechanisms underlying microbiome-assisted thermal resilience.

At the molecular level, our results suggest that the acclimated microbiome prompted the animals to adopt an energy-saving strategy in response to heat stress. Interestingly, this microbiome-mediated buffering effect appears to represent a general mechanism by which microbes protect their hosts, dampening stress responses irrespective of the specific stressor [37,70]. Our findings reveal a global inverse relationship between microbial community activity and host gene expression under thermal stress (Fig. 5), with three major pathways significantly downregulated in clams that received the thermally primed microbiome. The coordinated down-regulation of genes involved in sterol and cholesterol transport supports a model of metabolic adjustment, indicating a shift towards energy conservation or membrane remodelling, potentially through reduced cholesterol synthesis and storage. Notably, beneficial bacteria have been shown to influence host gene expression related to cholesterol metabolism across a wide range of animal taxa, generally resulting in lower cholesterol levels in conditions related to disease or stress [71,72]. Similarly, autophagy was suppressed, potentially representing another strategy to conserve cellular resources. This finding is consistent with observations in corals, where non-acclimated individuals exhibit stronger immune activation and apoptotic responses than acclimated ones [73]. At the same time, reduced autophagy could be part of a metabolic reprogramming response in which energy and nutrients are redirected towards other adaptive processes, such as storage regulation and membrane stabilisation [74]. This is in line with the signal we observed in relation to sterol regulation (Fig. S6), which may similarly reflect the host’s attempt to stabilize cell structure and function under thermal stress. Since priming involves prior exposure to heat, at least on the microbial side, this suppression may also reflect an adaptive rebound: a regulatory mechanism that avoids unnecessary self-digestion or overactivation of the autophagic process during repeated exposure to stress. The limitation of damage accumulation and preservation of tissue integrity were further supported by the upregulation of the replicative senescence pathway (Fig. 4). Emerging research shows that microbial metabolites can induce host senescence-like transcriptional programmes, thereby restricting the proliferation of damaged or stressed cells [75,76].

## Conclusion

Altogether, our results suggest that the thermally primed microbiome promotes cellular homeostasis not by eliminating stress altogether, but by helping the host maintain a more controlled, less damaging physiological state. Notably, unlike previous studies that used animals from clonal lines, our experimental animals varied in age and were not clonally derived, potentially introducing greater genetic diversity. This suggests that, under certain stress conditions, the adaptive influence of the microbiome may outweigh host developmental and genetic differences. These findings have significant biological and ecological implications, underscoring the importance of recognizing microbes as an integral component of eukaryotic physiology [77]. Furthermore, our results emphasise the importance of considering microbiomes when developing applied strategies aimed at enhancing organismal resistance. This concept has already gained momentum through global research efforts focused on coral reef restoration, where microbiome manipulation has shown great promise in improving coral resilience to ocean warming [78,79]. This phenomenon holds unprecedented theoretical and practical potential for increasing thermal tolerance across diverse organisms and offers promising applied solutions for aquaculture systems, where environmental stressors increasingly threaten sustainability and productivity. In this context, it is important to note that laboratory-based studies, including ours, necessarily involve a degree of ecological simplification in order to demonstrate direct links or associations, such as host–microbe interdependence. It is therefore critical to expand microbiome-based research to encompass a wider array of host species with the ultimate aim of informing novel approaches to conservation and environmental resilience.

## Supplementary Material

ycag059_Supplemental_Files

## Data Availability

Host RNA sequencing and 16S rRNA gene amplicon data are available in the NCBI database under BioProject accession PRJNA1292094. The *Ruditapes philippinarum* genome used for transcriptomics analysis has been deposited to the European Nucleotide Archive (ENA) under accession number GCA_982114075 (Project PRJEB108310). Custom R code used for differential expression gene calling and functional analyses is available at https://github.com/GEMMA-BCA/RNAseq-Analysis-2026.
