# Supplementary material for: Standing on the shoulders of microbes: microbiome thermal priming buffers the effects of heatwaves on clams by preventing stress overreaction
Source: ISME Commun. 2026 Mar 13;6(1):ycag059. doi: 10.1093/ismeco/ycag059 (PMC13077298; doi:10.1093/ismeco/ycag059)
Supplement: ycag059_Supplemental_Files [file ycag059_supplemental_files.zip › Supplementary Material Legends_0503.docx]

**Supplementary Figure 1.** Box plots show the Shannon diversity indices for each experimental group. Statistical comparisons were performed using the Kruskal–Wallis test followed by Dunn’s post-hoc pairwise comparisons limited to the selected biologically relevant contrasts. Adjusted p-values were corrected for multiple testing using the Benjamini–Hochberg (FDR) method. Box plots display the median (center line), the first and third quartiles (box limits), and whiskers extending to 1.5 × the interquartile range. Adjusted p-values are provided for the statistical comparisons between groups.

**Supplementary Figure 2.** Principal Coordinates Analysis (PCoA) at the ASV level based on Bray–Curtis dissimilarity, illustrating sample clustering according to treatment.

**Supplementary Figure 3.** Relative taxonomic composition of the microbiome at the genus level among treatment groups. Bar plot of significant genera (genera with relative abundance <0.1% are grouped as “Other”).

**Supplementary Figure 4.** Relative abundances of bacterial genera found to be significantly different between treatment groups based on DESeq2 analysis: (A) CM vs. PM, (B) CM_T vs. PM_T, and (C) CM_HW vs. PM_HW.

**Supplementary Figure 5.**  Principal Component Analysis (PCA) illustrating the distribution of clams transplanted with either a control (CM_HW) or thermally primed (PM_HW) microbiome and subjected to thermal challenge (experimental run 2). Data are plotted along the first (PC1) and second (PC2) principal components. Percentages in parentheses indicate the proportion of variance explained by each component.

**Supplementary Figure 6.** **Heatmaps of genes contributing to pathways significantly downregulated in PM_HW clams following GSEA analysis.** The heatmaps display the expression (log₂ fold change) of those genes included in the leading-edge subset of pathways associated with sterol-related (A) and autophagy-related (B) gene ontology (GO) pathways deemed significant following GSEA analysis in the comparison between clams transplanted with a thermally-primed microbiome (PM_HW) and with control microbiome (CM_HW), both subjected to thermal challenge. Each row represents a gene, and color intensity corresponds to its log₂ fold change, with negative values indicating reduced expression in PM_HW clams. White cells indicate that the gene is not present in the pathway.

**Supplementary Figure 7.** Inter-omics correlation presented by sample scatterplots displaying the two components of each dataset (microbiome and host transcriptome). Samples are coloured by treatment (PM_HW, CM_HW clams) and 95% confidence ellipse plots are represented.

**Supplementary Figure 8.** Loading plot showing the contribution of each variable to Component 1 for both datasets. Variables are ordered from bottom to top by the absolute value of their loading coefficients.

**Supplementary Table 1.** Details of the samples and associated metadata included in the RDA analysis (Treatment, Time, Temperature), along with the number of reads obtained from 16S rRNA sequencing retained after quality control filtering.

**Supplementary Table 2.** Differentially expressed genes identified through transcriptomic analysis. The table presents genes exhibiting significant expression differences between the experimental groups PM_HW and CM_HW. For each gene, the following information is provided: gene name (GeneID), NCBI accession number, functional annotation, log fold change (logFC), p-value, and false discovery rate (FDR). Positive logFC values indicate upregulation in the experimental group relative to the control, whereas negative values indicate downregulation.

**Supplementary Table 3.** List of amplicon sequence variants (ASVs) detected in PM_HW and CM_HW clams and associated taxonomic classification.

**Supplementary Table 4.** List of the 26 amplicon sequence variants (ASVs) identified as negatively correlated with the host transcriptomic response based on the DIABLO integrative analysis.

**Supplementary Table 5.** Genes identified through DIABLO mixOmics analysis with their corresponding functional annotations.
