# Supplementary figures and images for: Standing on the shoulders of microbes: microbiome thermal priming buffers the effects of heatwaves on clams by preventing stress overreaction
Source: ISME Commun. 2026 Mar 13;6(1):ycag059. doi: 10.1093/ismeco/ycag059 (PMC13077298; doi:10.1093/ismeco/ycag059)

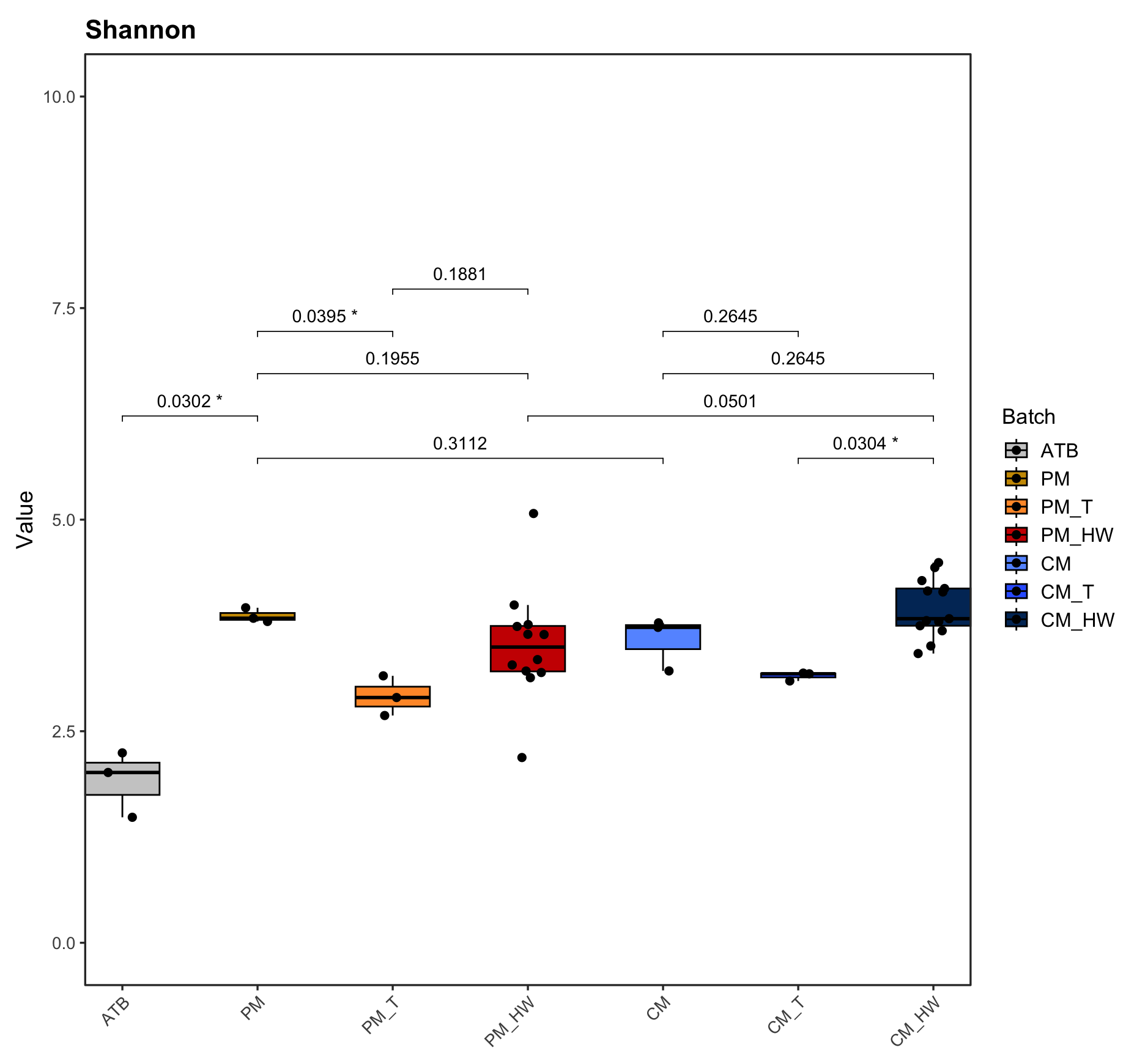

Supplement: ycag059_Supplemental_Files [file ycag059_supplemental_files.zip › Fig_S1_new.png]

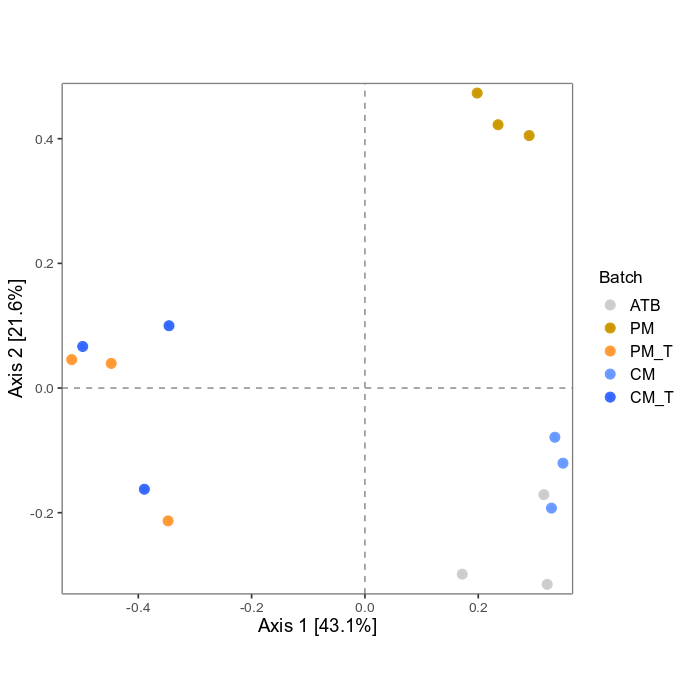

Supplement: ycag059_Supplemental_Files [file ycag059_supplemental_files.zip › Fig_S2.png]

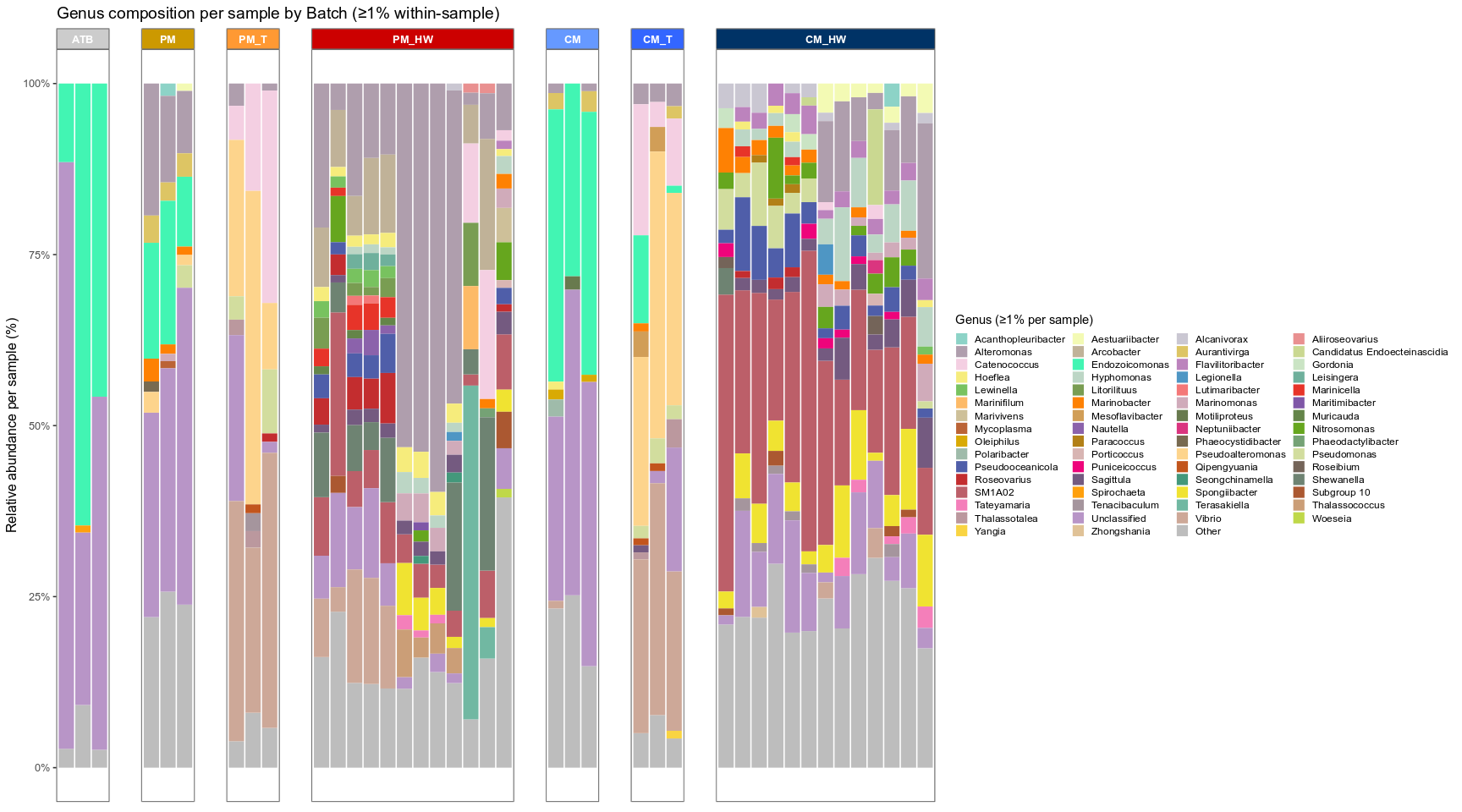

Supplement: ycag059_Supplemental_Files [file ycag059_supplemental_files.zip › Fig_S3.png]

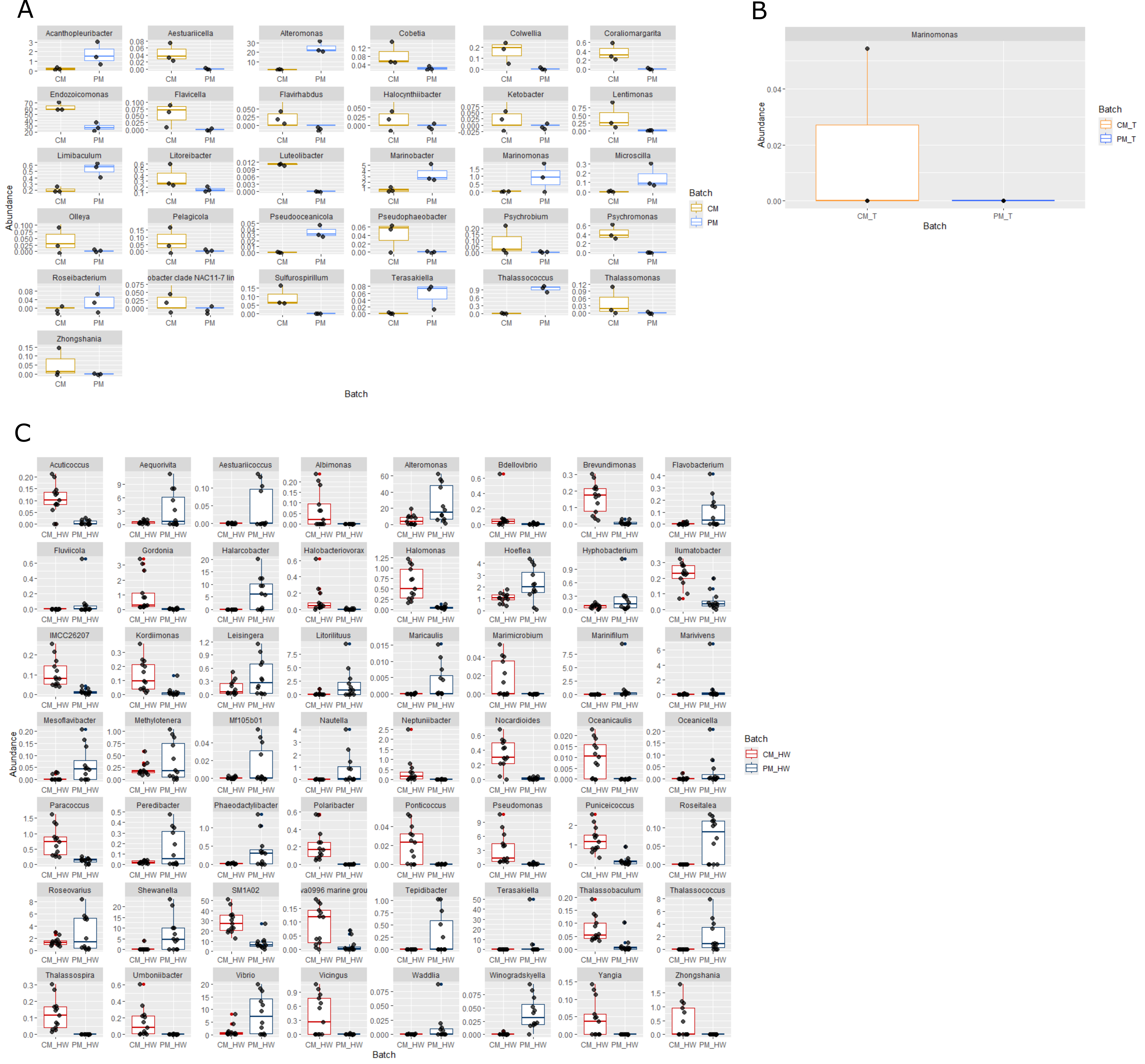

Supplement: ycag059_Supplemental_Files [file ycag059_supplemental_files.zip › Fig_S4_1901.png]

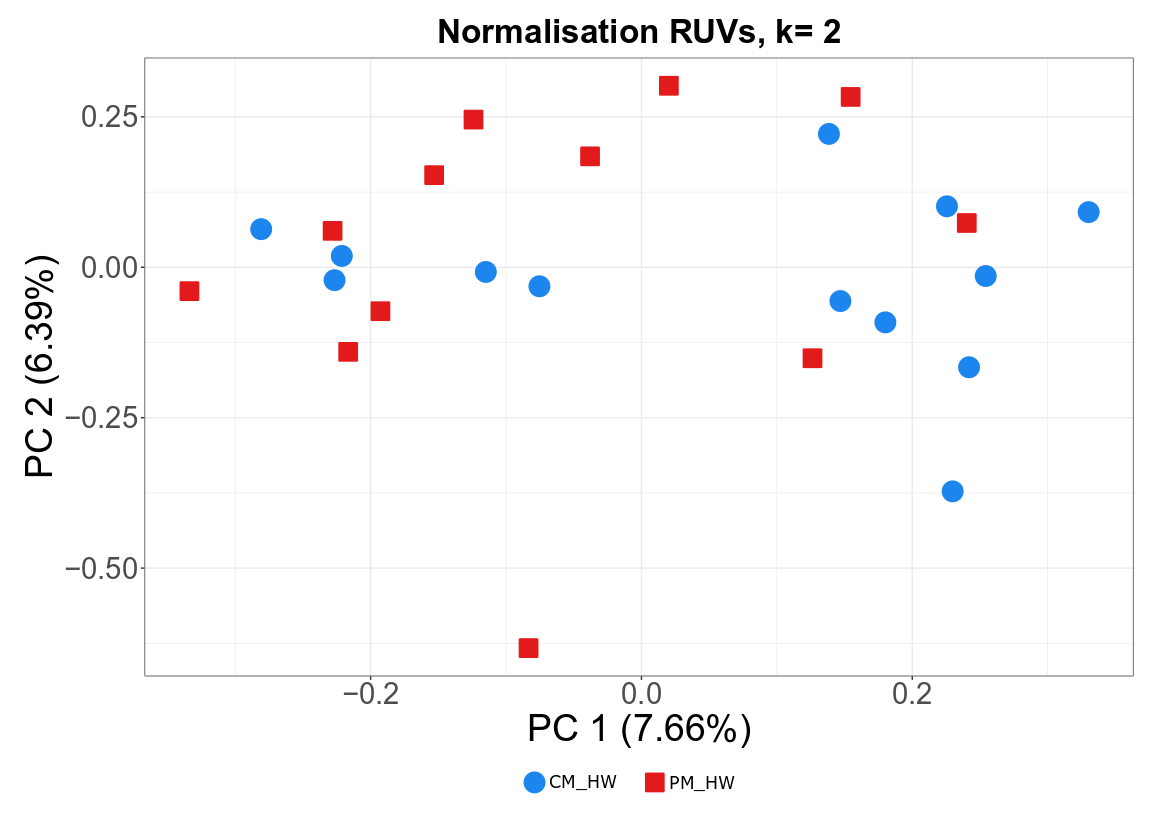

Supplement: ycag059_Supplemental_Files [file ycag059_supplemental_files.zip › Fig_S5.png]

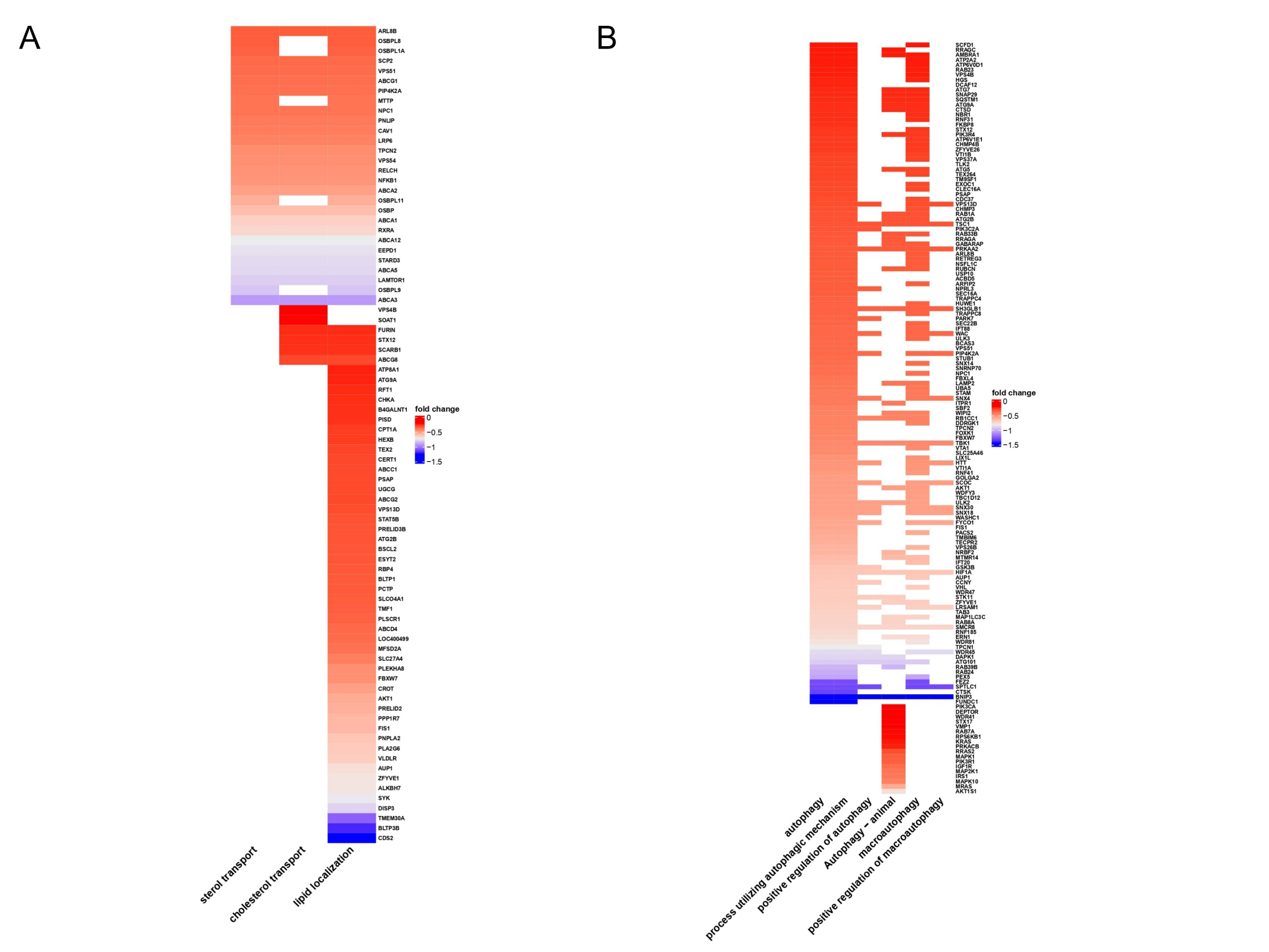

Supplement: ycag059_Supplemental_Files [file ycag059_supplemental_files.zip › Fig_S6.png]

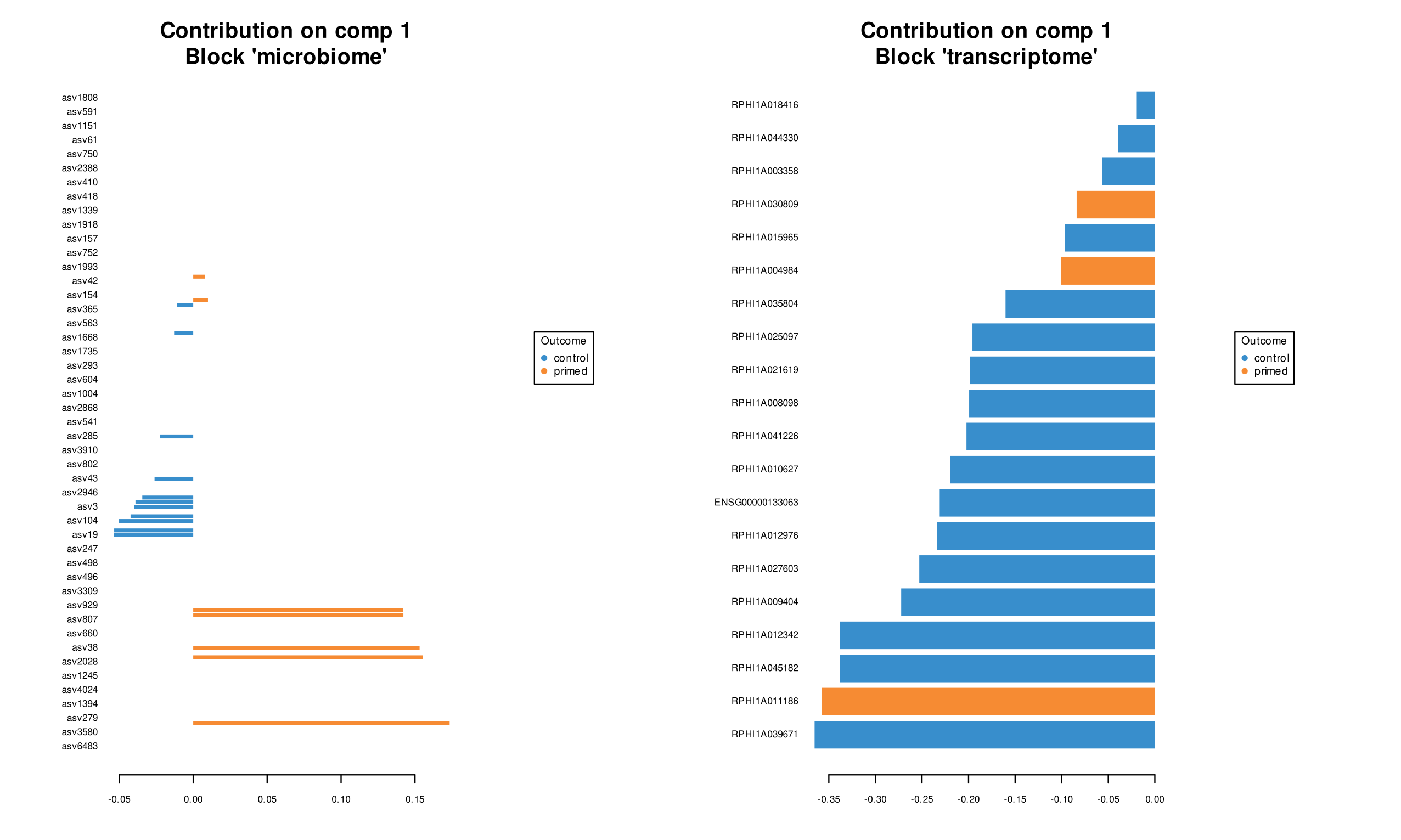

Supplement: ycag059_Supplemental_Files [file ycag059_supplemental_files.zip › Fig_S8.png]

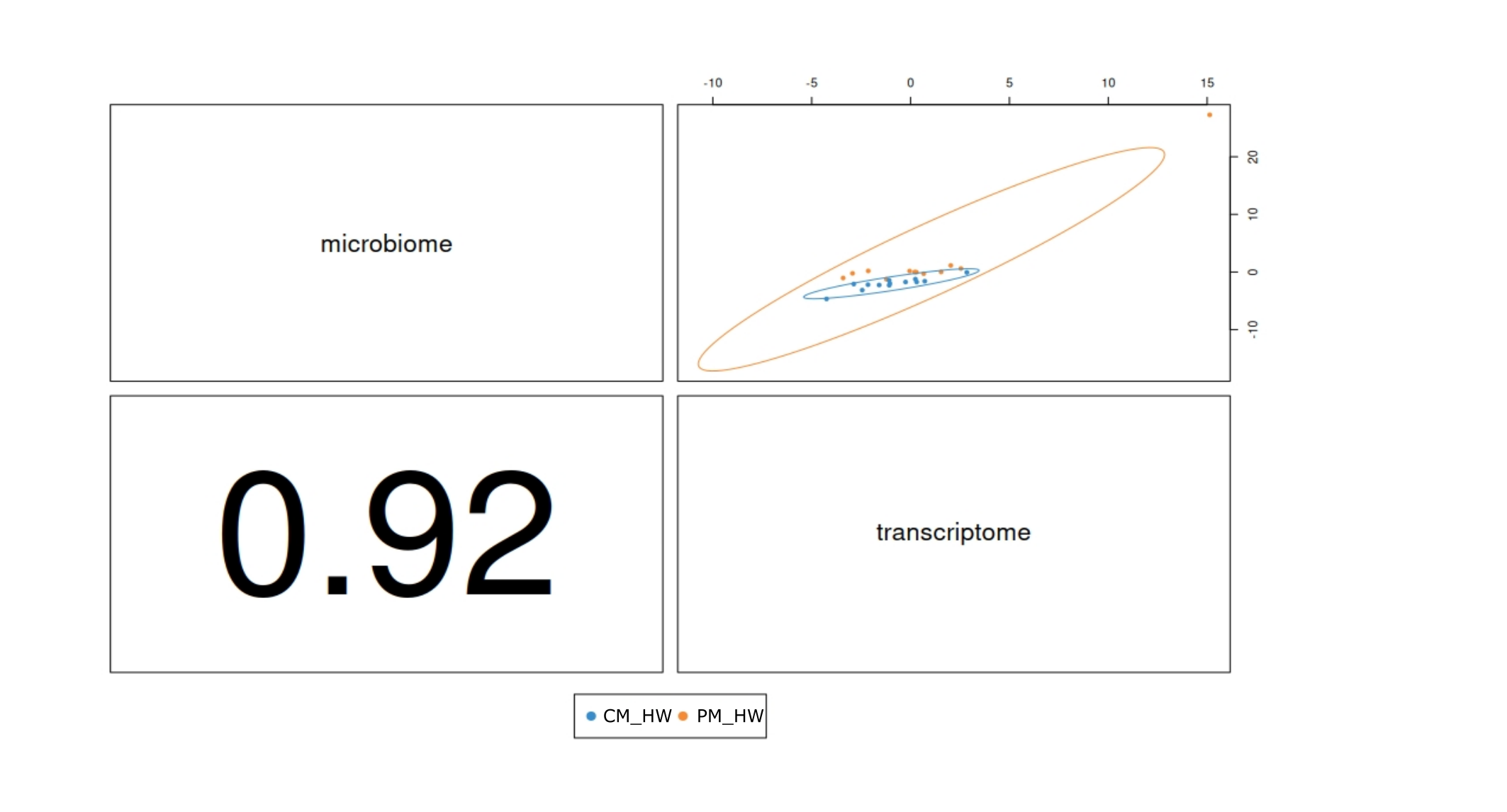

Supplement: ycag059_Supplemental_Files [file ycag059_supplemental_files.zip › FigS7.png]
